# The Interconnectedness of Euglycemic Diabetic Ketoacidosis With Concomitant Thyroid Storm: A Case Report

**DOI:** 10.7759/cureus.58696

**Published:** 2024-04-21

**Authors:** Kevin D Strawn, Keith W Davis

**Affiliations:** 1 Internal Medicine, Billings Clinic, Billings, USA

**Keywords:** sglt-2 inhibitor, endocrine emergencies, gluconeogenesis, critical care and hospital medicine, thyroid-storm, euglycemic diabetoketoacidosis

## Abstract

Sodium-glucose transport protein 2 inhibitors (SGLT2i) are becoming commonplace in many chronic diseases: type 2 diabetes mellitus, heart failure, and chronic kidney disease. We present the case of a 65-year-old male with a history of type 2 diabetes who had been on an SGLT2i for over 12 months and was found to have euglycemic diabetic ketoacidosis (eDKA) occurring concurrently with a thyroid storm. This case report illustrates a unique scenario of two endocrine emergencies occurring simultaneously.

## Introduction

SGLT2i are medications that act on the renal proximal convoluted tubule to reduce reabsorption of glucose, promoting glucose excretion in the urine. A side effect of SGLT2i therapy, seen with increasing frequency as more indications for SGLT2i become available, is eDKA, where a patient is euglycemic (blood glucose less than 250 mg/dL), has severe metabolic acidosis (pH less than 7.3, bicarbonate less than 18 mEq/L), and has ketonemia [1].

A thyroid storm is defined as an acute, life-threatening complication of hyperthyroidism that presents with multi-organ involvement and carries a mortality rate of 8-25% [2]. A thyroid storm is diagnosed clinically, with common symptoms including, but not limited to, fever, tachycardia, gastrointestinal symptoms, and neurological changes. Confirmatory thyroid tests commonly show high levels of free triiodothyronine (T3) and thyroxine (T4) with low thyroid-stimulating hormone (TSH). Concurrent thyroid storms and eDKA are rarely described in the literature, making this a unique presentation of two disease states that require somewhat contradictory management.

## Case presentation

A 65-year-old male with a history of type 2 diabetes mellitus, iron deficiency anemia, and hyperlipidemia presented to the emergency department at a critical access hospital with four days of nausea, vomiting, fatigue, increased lethargy, rigors, and diarrhea. Upon arrival at their hospital, he was diagnosed with eDKA as well as an elevated anion gap metabolic acidosis, treated with 3 liters of fluid resuscitation, and sent to the tertiary care center for further stabilization and management. Upon arrival at the tertiary care hospital, vital signs were significant for temperature of 38.0°C (100.4°F), blood pressure of 161/74, heart rate of 137, respiratory rate of 24, and maintaining oxygen saturations of 97% on room air. Weight on admission was 77.8 kg, with approximately 6 kg of weight loss in the last year. The patient was in mild acute distress, had a tachycardic heart rate with a regular rhythm, was noted to be warm to the touch, and had facial erythema present. He was alert and oriented to himself, the date, and the reason for the presentation but fell asleep frequently during the exam.

The patient's initial laboratory analysis was significant for a metabolic acidosis with a pH of 7.04, PCO2 of 22, and bicarbonate of six, resulting in an elevated anion gap of 22. The complete blood count was significant for leukocytosis (white count of 14.5k), hemoglobin 16.8, hematocrit 49.8, and platelets 269. Urinalysis revealed glucosuria with ketones present; ketones were also present in the serum. All other electrolytes were within normal limits, including a creatinine of 1.03. TSH was undetectable, free T3 was elevated at 7.64, T3 total elevated at 2.14, and T4 free elevated at 2.89. Thyroid antibody analysis was positive for an elevated thyroid-stimulating antibody, which, in combination with elevated T3/T4, was consistent with Graves’ thyroiditis. The initial electrocardiogram showed sinus tachycardia at a rate of 137, with nonspecific ST and T wave abnormality changes. Computed tomography (CT) scans of the head, chest, abdomen, and pelvis showed no acute findings. Ultrasound of the thyroid revealed a heterogeneous appearance of the thyroid gland without any definitive nodules.

The review of the medical records indicated that the patient had been on empagliflozin for over 12 months before this presentation. He had seen his primary care physician two months prior with no new complaints and no changes to his medications.

His Burch-Wartofsky Point Scale for Thyrotoxicosis was calculated at 60, highly suggestive of a thyroid storm (Table 1). 

**Table 1:** Burch-Wartofsky Point Scale for Thryotoxicosis

Criteria	Point scale	Patient Score - based on physical examination findings and vitals
Thermoregulatory dysfunction: temperature (°F)		
99.0-99.9	5	
100.0-100.9	10	10
101.0-101.9	15	
102.0-102.9	20	
103.0-103.9	25	
≥104.0	30	
Central nervous system dysfunction		
Absent	0	
Mild (agitation)	10	
Moderate (delirium, psychosis, extreme lethargy)	20	20
Severe (seizures, coma)	30	
Gastrointestinal-hepatic dysfunction		
Absent	0	
Moderate (diarrhea, nausea/vomiting, abdominal pain)	10	10
Severe (unexplained jaundice)	20	
Cardiovascular dysfunction		
Heart rate (beats/minute)		
<90	0	
90-109	5	
110-119	10	
120-129	15	
130-139	20	20
≥140	25	
Congestive heart failure		
Absent	0	0
Mild (pedal edema)	5	
Moderate (bibasilar rales)	10	
Severe (pulmonary edema)	15	
Atrial fibrillation		
Absent	0	0
Present	10	
Consequence	Total score	Total score
Unlikely thyroid storm	<25	
Impending storm	25-44	
Thyroid storm	>45	60

Once a thyroid storm was confirmed via laboratory confirmation in the emergency department, he was treated with 300 mg IV of hydrocortisone, methimazole 10 mg, and propranolol 60 mg. The patient was subsequently transferred to the intensive care unit for an insulin drip for his eDKA, as well as aggressive management given his thyroid storm. Following this management, there was significant clinical improvement in the patient’s vital signs, physical exam, and symptoms.

His empagliflozin was held during the admission and subsequent discharge as the suspected culprit of his eDKA, as no other precipitating event was identified. He was eventually able to be discharged home on methimazole monotherapy for Graves’ thyroiditis. Subsequent laboratory analysis after discharge showed a hemoglobin A1C of 9.0, a C-peptide of 3.7, and an insulin level of 19.5 with negative insulin autoantibodies (IAA), zinc transporter eight antibodies (ZnT8), islet cell antibodies (ICA), glutamic acid decarboxylase antibodies (GAD65), and insulinoma-associated-2 autoantibodies (IA-2A), consistent with the patient's diagnosis of type 2 diabetes mellitus. Had their C-peptide and insulin levels been depressed or any of the autoantibodies resulted in a positive result, this would have been more consistent with type 1 diabetes or latent autoimmune diabetes in adults (LADA) instead of eDKA.

## Discussion

SGLT2i was first introduced as a treatment for type 2 diabetes mellitus. A life-threatening side effect seen with patients on SGLT2i is euglycemic diabetic ketoacidosis, which has an incidence of approximately 0.1% [1], occurring in 5-12% of type 1 diabetics and <0.1% of type 2 diabetics [1]. The rates of EDKA with SGLT2i in clinical trials are 0.2-0.6 per 1000 patient-years at 10 and 25 mg doses of empagliflozin, respectively [3]. Other listed side effects include genital and urinary tract infections, increased risk of leg and foot amputations, and increased bone fracture risk [4]. SGLT2i is now recommended for heart failure patients [5] as well as chronic kidney disease patients with or without diabetes [6]. Specifically, SGLT2i is recommended for patients with 1) heart failure with reduced ejection fraction (1A recommendation) [5]. 2) Heart failure with a moderately reduced ejection fraction (2A recommendation) [5]. 3) Heart failure with preserved ejection fraction (2A recommendation) [5]. 4) Patients with chronic kidney disease (CKD), without diabetes, and a urine albumin-creatinine ratio >230 mg/g (2B recommendation) to reduce the risk of progression of CKD [6].

From 2015 to 2020, there was a 101% increase [7] in prescriptions of SGLT2i in the United States, two years before the 2022 AHA/ACC/HFSA guidelines recommended them for treatment of heart failure. Recent studies on SGLT2i in 2020 (DAPA-CKD [8]) and 2023 (EMPA-KIDNEY [9]) showed a lower risk of progression of chronic kidney disease as well. In 2020, the prevalence of heart failure in the United States was 6.7M (2.03%) [10] but is projected to rise to 8.5M (2.39%) by 2030 [10]. Globally, chronic kidney disease is projected to rise to the fifth highest cause of years of life lost by 2040, outpacing diabetes mellitus in terms of years of life lost [11]. In the United States alone, patients with type 2 diabetes mellitus are expected to rise to 54.9M by 2030 [12]. As such, medical providers are likely to see an increased patient base prescribed SGLT2i, thus making the aforementioned side effects more common in hospitals and clinics.

Our case represents a conundrum for the medical professionals who take care of patients with acute, life-threatening illnesses. In managing eDKA, fluids, insulin, and glucose are employed as necessary. The first step is to stop the offending agent, in our case, the SGLT2i, fluid replacement with serial monitoring of electrolytes as the typical eDKA patient is significantly volume depleted, and a continuous insulin infusion to suppress the formation of additional ketones by decreasing gluconeogenesis and glycogenolysis [13]. Once blood glucose concentrations have been reduced to less than 250 mg/L, a D5W solution should be started to enhance clearance and reduce the production of ketone bodies [1]. Monitoring of ketones and electrolytes should be done every two hours until blood ketones are less than 0.6 mmol/L and electrolytes are stabilized [1].

A concurrent thyroid storm requiring emergent treatment significantly complicates the management of eDKA. In general, once a thyroid storm is identified, patients are given a beta blocker to achieve a heart rate between 60 and 80 beats per minute, a thionamide to reduce thyroid hormone synthesis, and finally, glucocorticoids (loading dose followed by maintenance dosing) to reduce T4-to-T3 conversion and treat associated limited adrenal reserve. Specifically, beta blockers and steroids are not typically recommended as standard of care in eDKA.

Beta-blockers, either specific or non-specific, decrease both the rate and contractility of the heart at B­1 and B2 receptors in the myocardium, with some acting on A1 receptors as well. In a patient with already decreased intravascular volume from eDKA, their tachycardia represents a normal physiologic response to said hypovolemia, maintaining what little reserve they may have in cardiac output. In general, this physiologic response (tachycardia) should not be suppressed in eDKA. However, in the case of a thyroid storm, beta-blockers are a mainstay of treatment, reducing the beta-adrenergic effects that elevated levels of thyroid hormone cause. Fortunately, in our patient’s case, his cardiac reserve was high enough that the reduction in heart rate did not cause hypotension, likely due to volume repletion received at the outside hospital before receiving his propranolol. Likely, if our patient had less cardiac reserve or more severe hypovolemia, he may have required vasopressors after receiving the propranolol.

Second, glucocorticoids, typically hydrocortisone in thyroid storm, are not recommended in any type of diabetic ketoacidosis due to their mechanism of action. Hydrocortisone, a steroid with a roughly equal glucocorticoid and mineralocorticoid mechanism, is metabolized to cortisone by 11B-hydroxysteroid dehydrogenase, an intracellular enzyme [14]. Cortisol acts on many receptors but specifically acts on the liver to increase gluconeogenesis and decrease insulin sensitivity, the pancreas to inhibit insulin in B-cells as well as increase glucagon secretion in A-cells, increase lipolysis in lipid cells, decrease glucose uptake in muscle cells, and decrease capillary recruitment [15]. Although these are all important functions when treating thyroid storms, they become problematic when treating eDKA simultaneously.

As such, when these two endocrine emergencies occur simultaneously, treatment for one (thyroid storm) can lead to unintended consequences in the second (eDKA) and may precipitate the need for ICU-level care for close monitoring.

## Conclusions

As the population trends towards increasing numbers of patients on SGLT2i, given indications outside of just type 2 diabetes mellitus, eDKA will be expected to become more commonplace in intensive care units, emergency departments, and hospital floors. Although a relatively rare occurrence, a thyroid storm is another endocrinologic emergency with high mortality rates. Clinicians treating these endocrinologic emergencies, concurrently should be aware of the interconnectedness of the disease processes and treatments and be prepared for unintended outcomes when attending to these patients, as the treatment for one may be detrimental to the treatment of the other.
